# COG1410 Alleviated Chronic Sleep Deprivation-Induced Memory Loss by Regulating Microglial Phagocytosis and Inhibiting Hippocampal Inflammation

**DOI:** 10.1021/acschemneuro.5c00214

**Published:** 2025-07-09

**Authors:** Peng Wu, Chao Fu, Min Chen, Fanchan Wu, Wanyou He, Qichen Luo, Hanbing Wang, Yalan Li

**Affiliations:** † Department of Anesthesiology, Jinan University First Affiliated Hospital, 613# The West of Huangpu Avenue, Tianhe District, Guangzhou 510630, Guangdong, China; ‡ Department of Anesthesiology, The Second Affiliated Hospital of Guangzhou University of Chinese Medicine, 111# Dade Road, Yuexiu District, Guangzhou 510120, Guangdong, China; § Department of Cardiology, Guangdong Cardiovascular Institute, Guangdong Provincial Key Laboratory of Prevention and Treatment of Coronary Heart Disease, Guangdong Provincial People’s Hospital (Guangdong Academy of Medical Sciences), Southern Medical University, 106# Zhongshan Second Road, Yuexiu District, 510080 Guangzhou, China; ∥ Department of Anesthesiology, The First People’s Hospital of Foshan, 81# North of Ling Nan Road, Foshan 528000, Guangdong, China

**Keywords:** chronic sleep deprivation, memory loss, microglia, phagocytosis, COG1410, hippocampus

## Abstract

It is widely recognized that sleep loss harms healthy adults. Microglia-mediated synaptic pruning is active during sleep and contributes to memory consolidation. Here, we established a mouse model of sleep deprivation (SD) using a modified multiple-platform method (MMPM). Using western blotting, immunofluorescence, and Golgi-Cox staining, we found that SD increased microglial capacity for phagocytizing mature synapses and decreased the number of mature synapses, which affected long-term memory consolidation but did not affect working memory after SD. Further, we discovered that pretreatment with the APOE mimic peptide COG1410 could partially rescue SD-induced long-term memory consolidation. Regarding the mechanism, COG1410 alleviated SD-induced abnormal microglial phagocytosis and increased the number of mature synapses. Also, COG1410 promoted M2 polarization of microglia and reduced hippocampal inflammation caused by SD. However, whether the anti-inflammatory effect of COG1410 is related to the regulation of microglial phagocytosis still needs further study. Finally, we downregulated the expression of TREM2 in the hippocampus using small interfering RNA (siRNA), which reduced the protective effect of COG1410 in SD mice. In summary, COG1410 has the potential to prevent SD-induced memory consolidation impairment by maintaining microglial phagocytosis and anti-inflammation in the mouse hippocampus.

## Graphical abstract

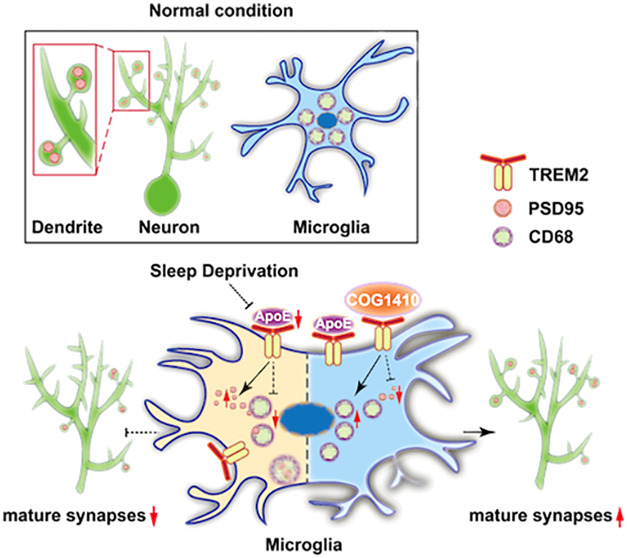

## 1 Introduction

Sleep loss has emerged as a significant public health concern in modern fast-paced societies. Epidemiological data have revealed alarming trends in sleep patterns worldwide. The 2014 Prevalence of Healthy Sleep Duration study indicates that one-third of American adults obtain less than 7 h of nightly sleep.^1^ Similar findings from China demonstrate that 27.1% of respondents in Guangdong Province reported insufficient sleep duration,^2^ falling substantially below the recommended minimum of 7–9 h per night for adults.^3^ This global pattern of sleep curtailment raises serious health concerns given the well-established associations between inadequate sleep and various physical and mental disorders.^4^ It is reported that only one night of sleep loss would induce the brain’s burden of amyloid-β­(Aβ),^5,6^ even, chronic sleep deprivation could result in memory disruption of laboratory animals^7,8^ or directly cause the death of experimental animals.^9,10^ Hence, enough sleep is necessary for most animals and human beings. Nowadays, an increasing number of people have to sacrifice sleep to confront various kinds of survival pressures, and it is somewhat urgent to prevent them from the harm of sleep deprivation.

It is unfortunate that there is no efficient way to keep people away from memory impairment induced by sleep deprivation because of the unknown neuronal mechanisms between sleep deprivation and memory impairment. The hippocampus is an important region in memory. During sleep, the reactivation of neurons in the hippocampal circuit highlights the crucial role of sleep in spatial memory related to the hippocampus.^11^ Within the hippocampus, synapses are considered as the basis of memory, which are constantly formed and eliminated. Previous evidence showed that sleep could affect the number and strength of synapses.^12–14^ As proper synaptic architecture constitutes the foundation of synaptic plasticity,^15,16^ recent experimental interventions using optogenetic suppression of hippocampal long-term potentiation (LTP) during sleep have been shown to significantly impair postsleep memory consolidation.^17^ Current research highlights the essential role of microglial activity in mediating synaptic plasticity within the central nervous system (CNS).^18,19^ These resident immune cells perform dual functions: (1) phagocytic pruning of redundant synapses to refresh hippocampal memory traces,^20,21^ and (2) secretion of neurotrophic factors such as brain-derived neurotrophic factor (BDNF) to modulate synaptic plasticity,^22,23^ Thus, maintaining microglial function in the hippocampus may contribute to synaptic plasticity and memory consolidation.

The triggering receptor expressed on myeloid cells-2 (TREM2) is a specific membrane receptor expressed on monocyte–macrophage lineage, including microglia.^24,25^ TREM2 mediates critical microglial functions encompassing phagocytic activity, lipid metabolism regulation, cellular survival maintenance, and anti-inflammatory responses.^25^ Particularly relevant to neural circuit refinement, TREM2 has been implicated in synaptic pruning and neural network optimization.^18,26^ Experimental evidence demonstrates that TREM2 deficiency in adult mice significantly impairs hippocampal synaptic plasticity,^27^ while emerging research indicates altered TREM2 expression patterns following chronic sleep deprivation,^28,29^ Building upon these findings, we propose a mechanistic hypothesis: TREM2 ligands may orchestrate hippocampal synaptic plasticity through microglia-mediated phagocytic regulation, thereby serving as critical modulators of memory consolidation processes.

COG1410, a novel synthetic peptide derived from the apolipoprotein E (APOE) receptor-binding domain, functions as a high-affinity APOE mimetic.^30,31^ COG1410 could combine with TREM2 and pass through blood–brain barrier.^23,30–33^ Preclinical studies have established its neuroprotective efficacy, with COG1410 administration shown to enhance motor function and spatial memory performance in murine models through the suppression of microglial activation and attenuation of neuronal apoptosis.^30^ In view of the above data, the present study investigates whether COG1410 relieves hippocampal-dependent memory loss in a murine model of sleep deprivation while elucidating the underlying mechanisms through which this peptide mediates neuroprotective effects.

## 2 Results

### 2.1 Chronic Sleep Deprivation Induced Long-Term Spatial Memory Loss, but Did Not Affect Working Memory after SD

To investigate the relationship between chronic sleep deprivation (SD) and spatial memory, we successively completed the Morris water maze test (MWM) and novel object recognition test (NORT), which are classic experiments for studying declarative memory. Mice in different groups were subjected to rigorous experimental processes (Figure 1A). After SD or no SD, we first performed the NORT to evaluate their working memory ability. As shown in Figure 1B–C, there was no difference between the control (CTRL) and SD groups in the percentage of time spent exploring the new object. Besides, the total distance traveled in the NORT of the two groups was close to each other, which suggested that the working memory of the experimental mice was unaffected by SD.

**Figure 1:**
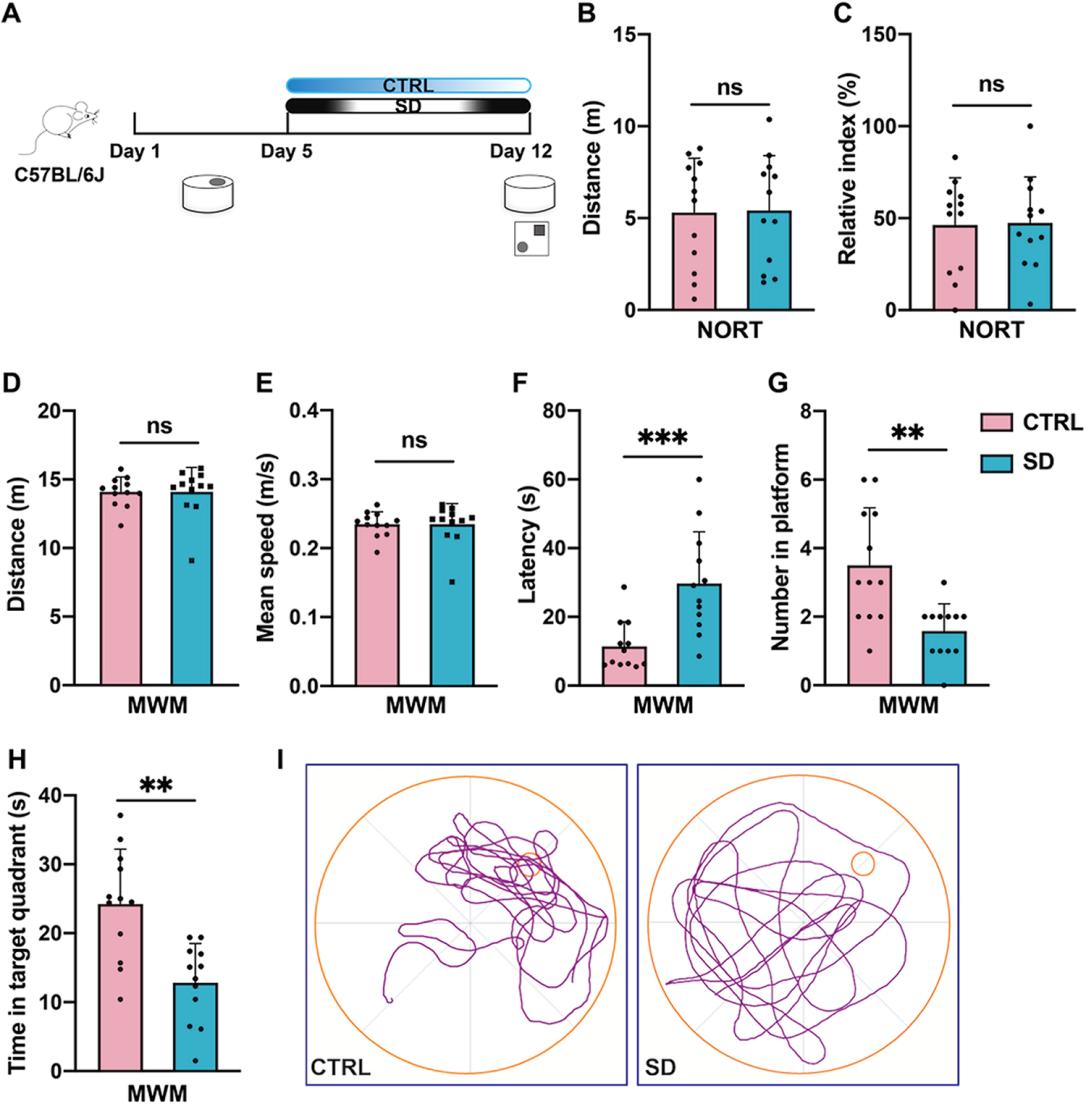
Chronic SD induced long-term spatial memory loss in adult mice. (A) Experimental design: sleep deprivation time and behavioral time point. (B) Chronic SD did not affect the motor ability of mice in the NORT (*n* = 12 per group, Student’s *t-*test, ns: no significance). **(C)** Chronic SD did not affect the RI of mice in the NORT (*n* = 12 per group, Student’s *t-*test, ns: no significance). (D) Chronic SD did not affect the swimming distance in the probe test (*n* = 12 per group, Student’s *t-*test, ns: no significance). (E) Chronic SD did not affect the swimming speed in the MWM (*n* = 12 per group, Student’s *t-*test, ns: no significance). (F) Chronic SD increased the latency of SD mice to the located platform in the probe test (*n* = 12 per group, Student’s *t-*test, *** *p* < 0.001). (G) Chronic SD reduced the number of SD mice on the platform in the probe test (*n* = 12 per group, Student’s *t*-test, ** *p* < 0.01). (H) SD mice spent less time in the target quadrant (*n* = 12 per group, Student’s *t-*test, ** *p* < 0.01). (I) Representative track plot of two groups of mice in the probe test.

Then, we used the MWM test to determine the impact of SD on long-term spatial memory. Results from the probe test showed that­(i) both swimming distance and swimming speed had no significant difference between the CTRL and SD groups (Figure 1D–E), which means that the two groups of mice had similar motor ability in the water maze; (ii) interestingly, compared to the CTRL group, mice in the SD group spent more time locating the platform zone (Figure 1F); (iii) in comparison, the number of platform crossings was reduced in the SD group (Figure 1G); and (v) mice that went through SD spent less time to explore in the target quadrant (Figure 1H). In a word, the above results indicated that chronic sleep deprivation would result in long-term spatial memory loss in mice.

### 2.2 Number of Mature Synapses in the Mice’s Hippocampus Was Reduced after Chronic SD

Working memory, also known as short-term memory, has a neural mechanism that is anatomically related to thalamic activity.^34^ Hippocampus is an important structure for long-term memory consolidation, where information from different pathways needs to be integrated and exported to the neocortex for memory storage.^11^ We first assessed the effects of chronic SD on hippocampal neurons. Our results of Nissl staining showed that the morphology and density of hippocampal neurons in experimental mice did not differ between the CTRL and SD groups (Figure 2A). Then, for evaluating the maturation of the synapse, we performed western blotting to examine the expression of postsynaptic density protein-95 (PSD-95), which is located in the postsynaptic membrane and is positively correlated with the number and size of dendritic spines.^35,36^ As shown in Figure. [Fig fig2]B–C, the level of PSD-95 was decreased significantly in the SD group compared to the CTRL group. Also, we used Golgi-Cox staining to assess the density of dendritic spines in hippocampal neurons (Figure 2D). Compared with mice in the CTRL group, an increase in dendritic spine density was observed in mice that underwent SD (Figure 2E). These results suggest that the loss of mature synapses and the excess of dendritic spines may be associated with SD-induced long-term spatial memory loss.

**Figure 2:**
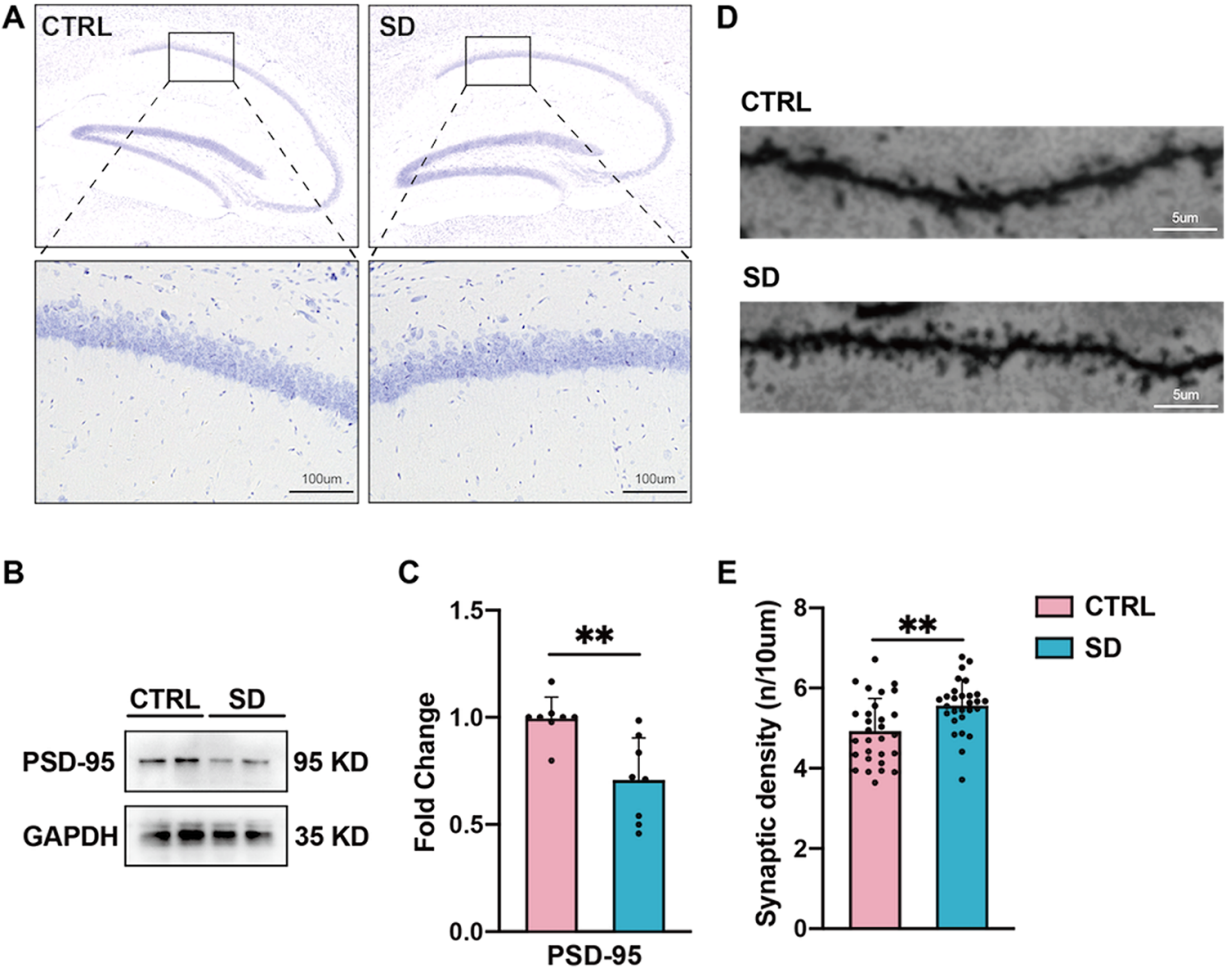
Chronic SD reduced mature synapses in the hippocampus. (A) Representative images of Nissl staining in CTRL and SD mice. There were no significant changes in the morphology or density of hippocampal neurons in CTRL and SD mice. (B) Representative images of the expression of PSD-95 in the hippocampus. (C). The ratio of PSD-95 proteins to the internal control in the control group was utilized as a reference for normalization. Compared with CTRL, SD reduced PSD-95 expression in the hippocampus. (*n* = 8 per group, Mann–Whitney test, ** *p* < 0.01). (D) Representative images of Golgi-Cox stained segments in the CTRL and SD groups. (E) Compared with CTRL mice, SD increases synaptic density in the hippocampus (*n* = 29 cells from 4 samples in CTRL; *n* = 28 cells from 4 samples in SD, Student’s *t-*test, ** *p* < 0.01).

### 2.3 SD Inhibited the Microglial Phagocytic Capacity

Since we found that PSD-95 was decreased in the hippocampal tissue of sleep-deprived mice, we wondered whether synaptic plasticity was changed by SD. In view of the great role that microglia play in synaptic plasticity, we then focused on looking for changes in microglia induced by SD. First, we used immunofluorescence staining to determine the number of hippocampal microglia. However, no significant difference was found in the count of Iba-1 positive cells in the hippocampus between the CTRL group and SD group (Figure 3A–B). According to recent studies^56^ that emphasized the important role of microglial phagocytosis in synaptic plasticity, we evaluated the expression of CD68, which is localized to microglial lysosomes and plays a role in phagocytic activity through immunofluorescence staining and western blotting. As shown in Figure 3C–D, compared with CTRL mice, the number of CD68-positive hippocampal microglia was decreased in sleep-deprived mice. Similarly, the hippocampal CD68 protein level is lower in the SD group than in the CTRL group (Figure 3E–F). That is to say, our results support that the phagocytic ability of hippocampal microglia in experimental mice is inhibited due to sleep deprivation.

**Figure 3:**
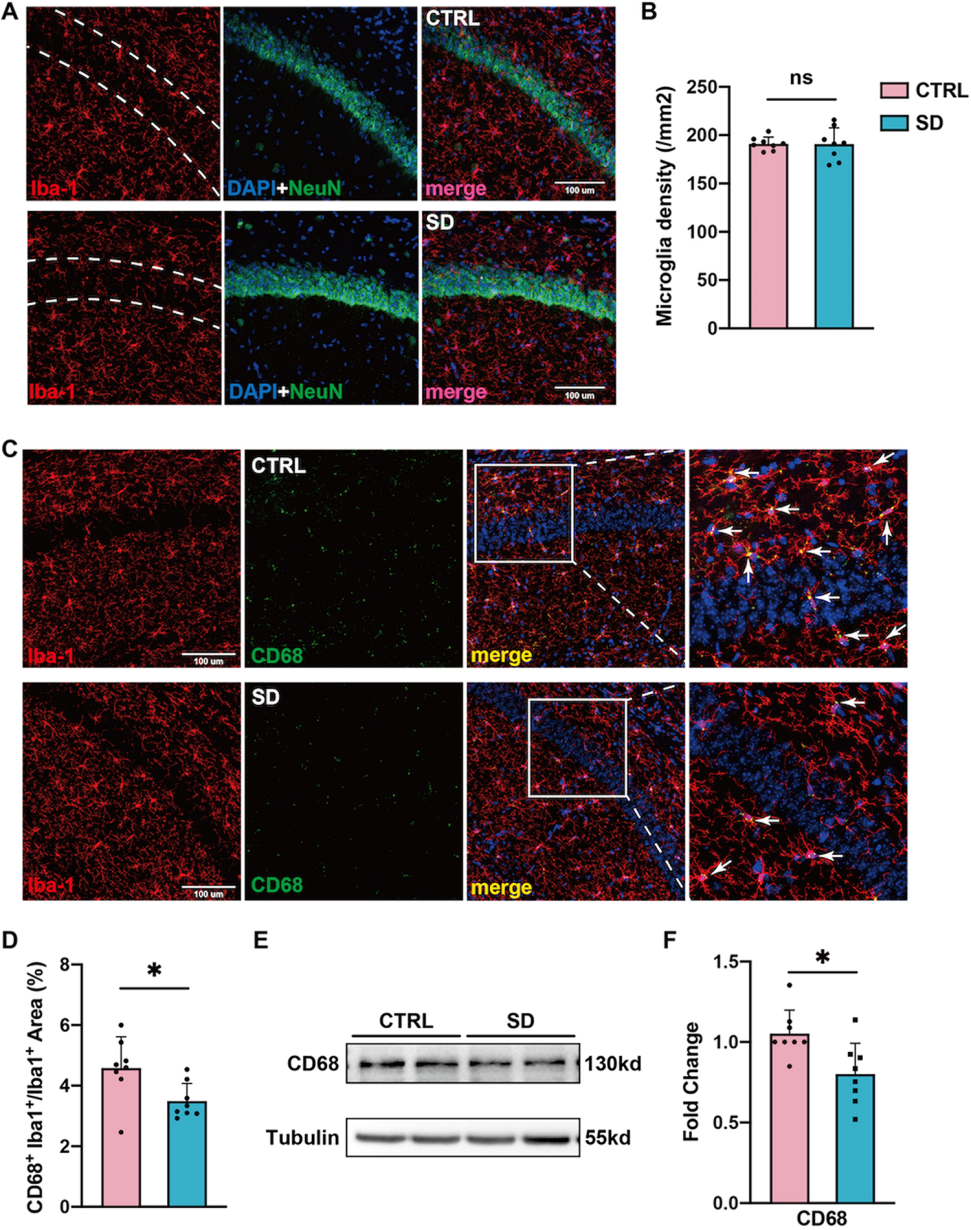
Chronic SD weakened microglia phagocytosis in SD mice. (A) Representative images of immunofluorescence staining of Iba-1­(red) and Neun (green) in the hippocampus. (B) The density of Iba-1^+^ cells in the hippocampus was not affected by SD (*n* = 8 per group, Welch’s *t* test, ns: no significance) (C) Representative images of immunofluorescence staining of Iba-1­(red) and CD68 (green) in the hippocampus. (D) SD reduced the CD68 and Iba1 double-positive areas in the hippocampus (*n* = 8 per group, Student’s *t-*test, * *p* < 0.05). (E) Representative images of the expression of CD68 in the hippocampus. (F) The ratio of CD68 proteins to the internal control in the control group was utilized as a reference for normalization. Compared with CTRL, SD reduced the expression of CD68 in the hippocampus (*n* = 8 per group, Student’s *t-*test, * *p* < 0.05).

### 2.4 Administration of COG1410 Relieved Mouse’s Long-Term Spatial Memory Loss Induced by Chronic SD

TREM2 is one of the specific membrane receptors located on the surface of microglia, which takes part in various activities of microglia, including migration, phagocytosis, and anti-inflammation. Apolipoprotein E (APOE) serves as a natural ligand for TREM2, functioning by activating TREM2. Moreover, our western blot experiment demonstrated that the level of TREM2 protein in the mouse hippocampus was slightly decreased in sleep-deprived mice, but no significant difference was observed when compared to mice in the CTRL group (Figure 4A–B). However, a statistical difference was observed when comparing the APOE protein level between the above two groups, as shown in Figure 4A,C. Hippocampal APOE expression was downregulated in mice undergoing SD. Therefore, we suggest that reduced expression of APOE is the cause of SD-induced impairment of microglial phagocytic capacity, which leads to deterioration of long-term spatial memory.

**Figure 4:**
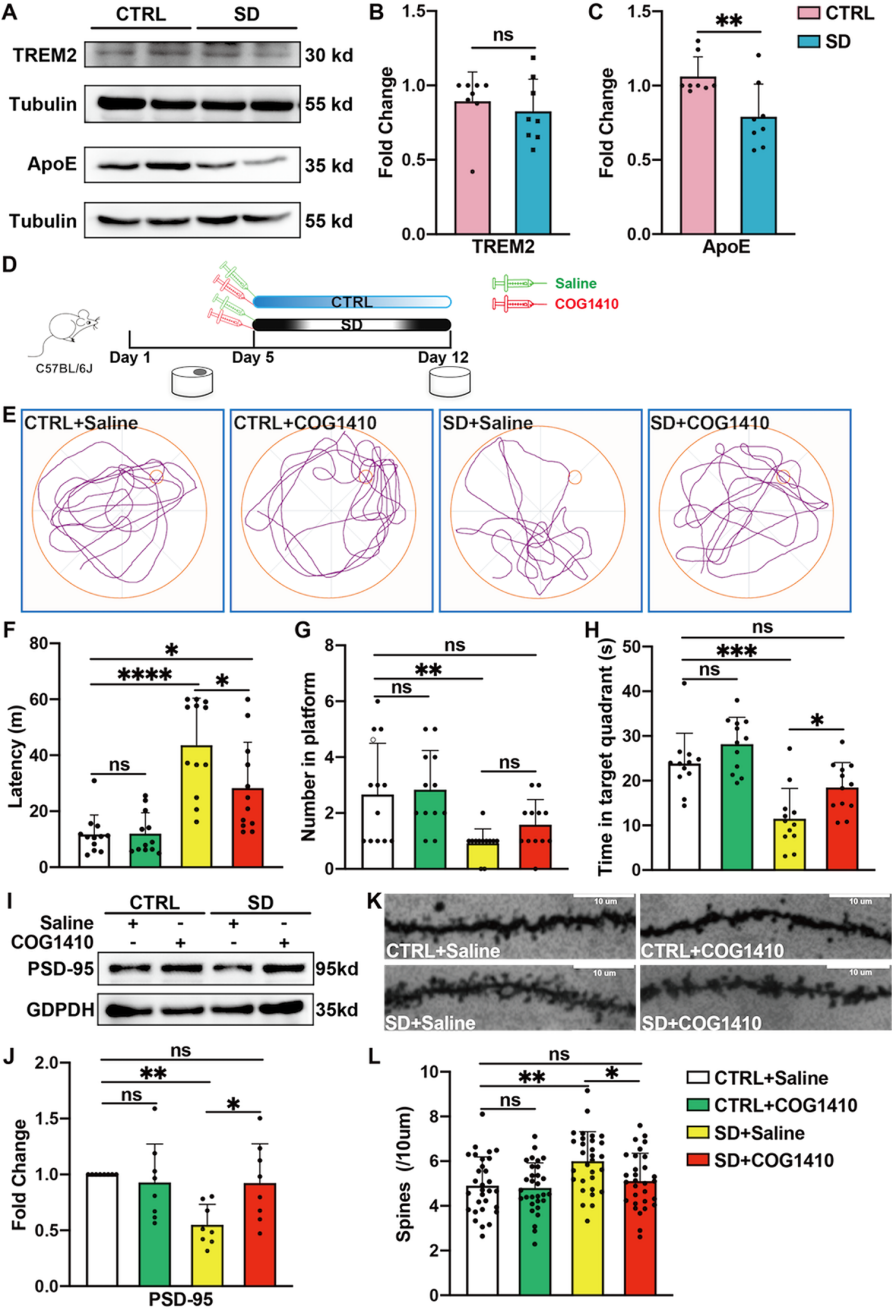
COG1410 partially alleviates chronic SD-induced spatial memory loss and its effects on synapses. (A) Representative images of the expression of TREM2 and APOE in the hippocampus. (B) The ratio of TREM2 proteins to the internal control in the CTRL group was utilized as a reference for normalization. SD did not affect the expression of TREM2 in the hippocampus (*n* = 8 per group, Mann–Whitney *t* test; ns: no significance) (C) The ratio of APOE proteins to the internal control in the CTRL group was utilized as a reference for normalization. Compared with CTRL, SD reduced the expression of APOE in the hippocampus (*n* = 8 per group, Mann–Whitney *t* test, * *p* < 0.05). (D) Experimental design: CTRL and SD mice were intraperitoneally injected with COG1410 (0.2 mg/kg) or saline 30 min before SD. (E) Representative track plot of four groups of mice in the probe test. (F) COG1410 increased SD induced reduction of latency in the probe test (*n* = 8 per group, one-way ANOVA test followed by Tukey’s multiple comparison post-test, ns: no significance, * *p* < 0.05, **** *p* < 0.0001). (G) COG1410 did not affect the number of SD mice on the platform in the MWM (*n* = 8 per group, nonparametric test, followed by Dunn’s multiple comparison post-test, ns: no significance). (H) COG1410 increased the time spent in the targeted quadrant of SD mice (*n* = 12 per group, one-way ANOVA test followed by Tukey’s multiple comparison post-test; ns, no significance, * *p* < 0.05, *** *p* < 0.001). (I) Representative images of the expression of PSD-95 in the hippocampus. (J) Compared with SD + Saline, COG1410 increased the expression of PSD-95 in the hippocampus. The ratio of the PSD-95 protein to the internal control in the CTRL + Saline group was utilized as a reference for normalization (*n* = 8 per group, one-way ANOVA test followed by Tukey’s multiple comparison post-test, ns: no significance, * *p* < 0.05, ** *p* < 0.01). (K) Representative images of Golgi-Cox-stained segments among the four groups. (L) Compared with SD + Saline, COG1410 reduced the synaptic density in the SD mice’s hippocampus (*n* = 30 cells from 4 samples per group, one-way ANOVA test followed by Tukey’s multiple comparison post-test; ns, no significance, * *p* < 0.05, ** *p* < 0.01).

In order to verify our hypothesis, we used COG1410, an APOE mimic peptide, to detect whether it can reverse SD-induced long-term spatial memory impairment. According to our experimental flowchart (Figure 4D), when mice finished spatial learning, half of the SD mice were chosen to receive COG1410 (0.2 mg/kg, i.p.), 30 min before daily SD. Half of the CTRL mice required the same administration at the same time. Then, on day 12, all mice would be compelled to undergo a probe test. A track plot of the 4 groups is shown in Figure 4E. The results of the probe test revealed that there was no statistical difference in the swimming distance or average swimming speed among the 4 experimental groups (Figure S1), which indicates that COG1410 did not affect the swimming ability of mice, whether they went through SD or not. Also, compared with the SD+Saline group, COG1410 significantly reduced the latency for sleep-deprived mice to find the platform region, while the use of COG1410 did not affect the latency of non-SD mice (Figure 4F). Besides, while there was no difference in the number of mice entering the platform among the four groups, COG1410 increased the amount of time the SD mice spent exploring the target quadrant (Figure 4G–H). Thus, the above results suggest that COG1410 might prevent SD-induced spatial memory loss. Furthermore, using western blotting to examine the expression of PSD-95 in hippocampal tissue among the above groups, the results showed that SD-induced downregulation of hippocampal PSD-95 was reverted by COG1410; however, COG1410 had no effect on PSD-95 expression in ordinary mic (Figure 4I–J). Besides, we also performed Golgi-Cox staining to detect the effect of COG1410 on the density of dendritic spines. As expected, COG1410 could reverse the SD-induced increase of dendritic spines, but had no effect on those in non-SD mice. The results of Golgi-Cox staining are shown in Figure 4K–L. To date, we are convinced that COG1410 protects against SD-induced long-term spatial memory loss.

### 2.5 COG1410 Partially Restored the Phagocytic Ability of Hippocampal Microglia that Can Deal with Excess Synapses Induced by SD

Considering that microglial activation may increase synaptic plasticity and maturity through the Akt/CREB/BDNF signaling axis,^23^ we tried to determine whether the alteration of PSD-95 protein and dendritic spine density resulting from COG1410 depends on the regulation of BDNF in microglia. The results of western blotting indicated that sleep deprivation reduces BDNF expression in mouse hippocampal tissue, but these processes could not be reversed by COG1410 therapy (Figure S2). Moreover, immunofluorescence staining showed that preadministration of COG1410 did not change hippocampal microglial density in the SD groups or non-SD groups (Figure 5A–B). Excitingly, the statistical results of CD68 expression by immunofluorescence staining demonstrated that COG1410 treatment increases the CD68-positive area in microglia of sleep-deprived mice compared to placebo treatment. However, in non-SD groups, with or without COG1410 administration, no significant difference was seen in the statistics of CD68-positive microglia using immunofluorescence staining (Figure 5C). Also, we reused western blotting to evaluate CD68 expression in mouse hippocampal tissue; not surprisingly, COG1410 alleviates the decrease of hippocampal CD68 proteins induced by SD, and COG1410 did not significantly influence the expression of CD68 proteins in mouse hippocampus when they were free of SD (Figure 5D). Altogether, there is a potential possibility that COG1410 may treat the impairment of the mouse hippocampus caused by SD by increasing the phagocytosis capacity of microglia.

**Figure 5:**
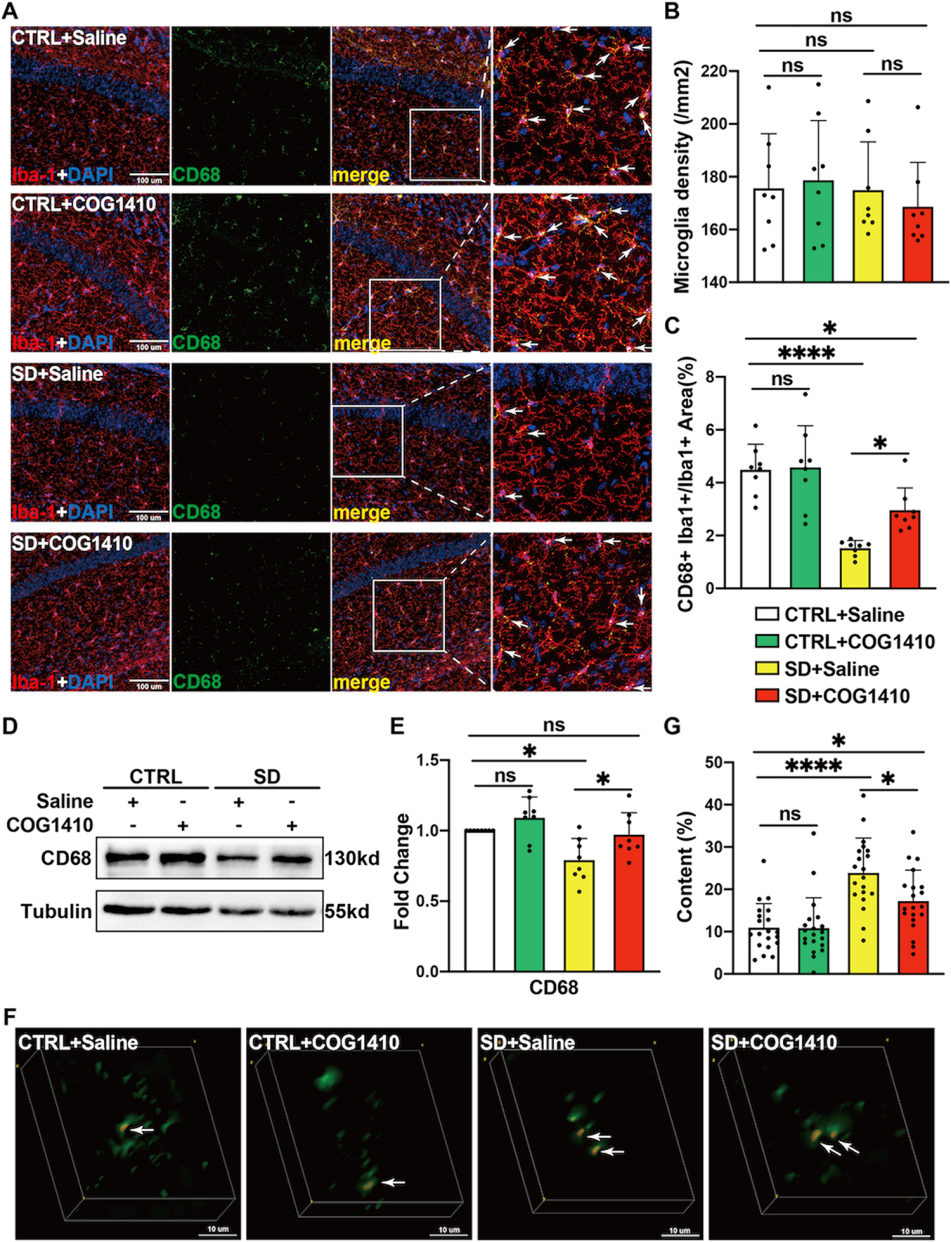
COG1410 enhanced microglia phagocytosis in SD mice (A) Representative images of immunofluorescence staining of Iba-1 (red) and CD68 (green) in the hippocampus. (B) COG1410 did not change the density of Iba-1^+^ cells in the hippocampus. (*n* = 8 per group, nonparametric test followed by Dunn’s multiple comparison post-test, ns: no significance). (C) COG1410 increased the microglial CD68^+^ area in the SD mice’s hippocampus (*n* = 8 per group, one-way ANOVA test followed by Tukey’s multiple comparison post-test, ns: no significance, * *p* < 0.05, **** *p* < 0.0001). (D) Representative images of the expression of CD68 in the hippocampus. (E) Compared with SD + Saline, COG1410 increased the expression of CD68 in the hippocampus (*n* = 8 per group, one-way ANOVA test followed by Tukey’s multiple comparison post-test, ns: no significance, * *p* < 0.05). (F) Representative images of the 3D colocalization of PSD-95^+^ (red) and CD68^+^ (green) double-positive puncta in the hippocampus. (G) Compared with SD + Saline, COG1410 reduced the ratio of the volume of PSD-95^+^ and CD68^+^ double-positive puncta to the volume of CD68^+^ puncta (*n* = 20 cells from 4 samples per group, one-way ANOVA test followed by Tukey’s multiple comparison post-test, ns: no significance, * *p* < 0.05, **** *p* < 0.05).

For further evidence of COG1410 affecting microglial phagocytic capacity, we reconstructed a 3D model of PSD-95 and CD68-positive cells based on immunofluorescence staining and calculated the costaining volume of PSD-95 and CD68 (Figure 5F), which revealed that COG1410 mitigated the SD-induced increase in the proportion of PSD-95 particles taken up in the microglial lysosomes (Figure 5G). Hence, we had enough evidence to say that COG1410 could restore microglial phagocytosis to mature synapses induced by SD in the hippocampus.

### 2.6 COG1410 Converts the Pro-inflammatory State of Microglia in the Hippocampus of Sleep-Deprived Mice

Microglial morphology dynamically varies with the polarization state. Therefore, in order to explore the effect of COG1410 on microglial function, we used Sholl analysis to analyze microglial morphology in the mouse hippocampus, and the brief process of this analysis is shown in Figure 6A. From this analysis, we found that no statistical difference in microglial morphology in the non-SD groups after COG1410 administration, irrespective of the longest projection length, mean projection length, total projection length, number of branch points, or number of terminals (Figure 6B–F). In sleep-deprived mice, although no significant difference was found in the longest projection length of microglia, COG1410 shortened the mean projection length and total process length of microglia compared with the placebo group (Figure 6B–D). Also, COG1410 decreased its number of branch points and terminals of microglia (Figure 6E–F). Besides, as shown in Figure 6G, SD reduced the complexity of hippocampal microglia, which could be imperfectly restored by COG1410.

**Figure 6:**
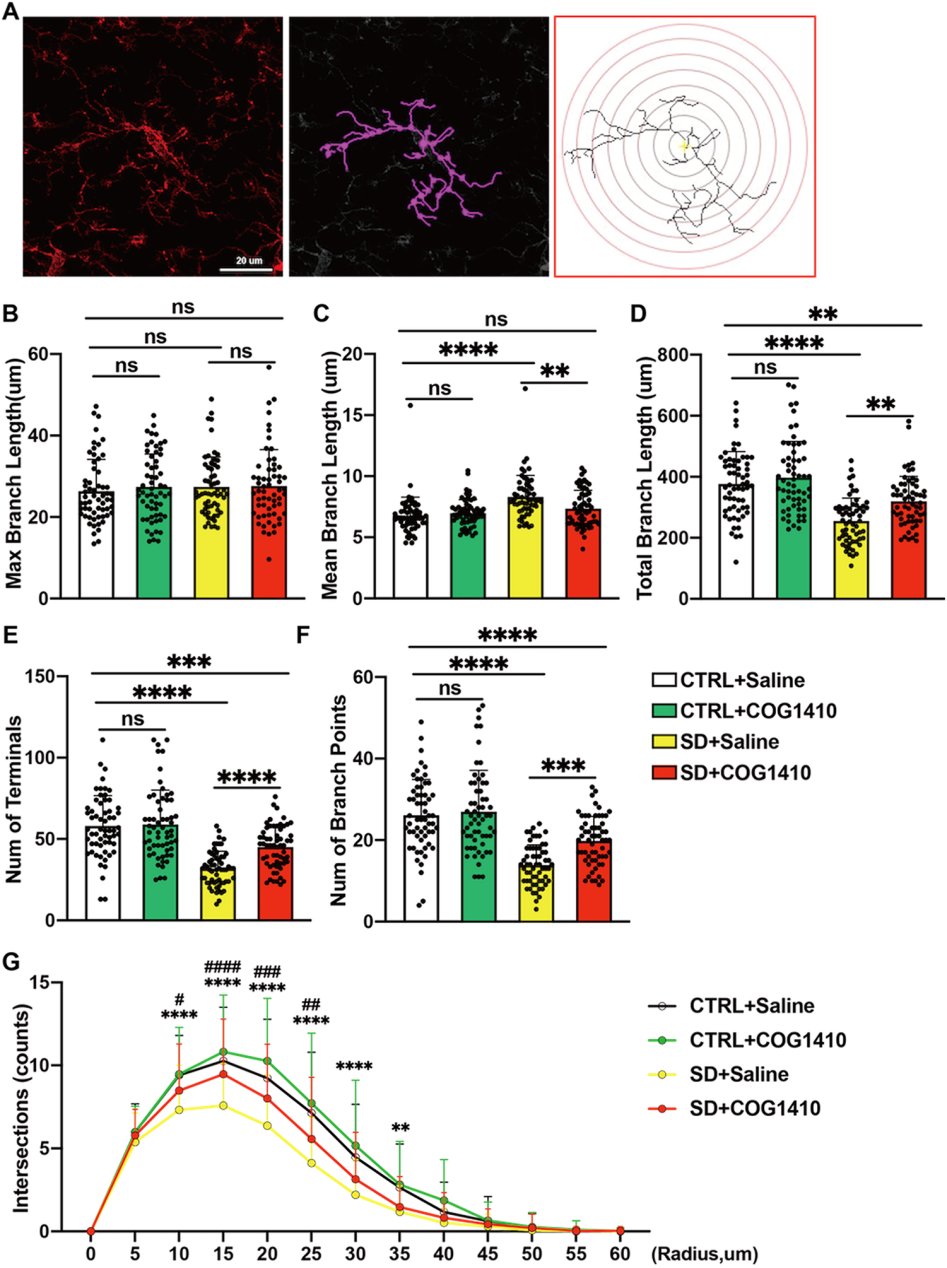
COG1410 attenuated chronic SD-induced morphological changes in hippocampal microglia. (A) Brief diagram of the Sholl analysis. Left: immunofluorescence staining of Iba-1; middle: magenta lines show the skeleton of microglia; right: quantitative analysis of the intersections of projections in the Sholl analysis. (B) No significant change was found in the maximum branch length of microglia in the four groups (*n* = 60 cells from 8 samples per group, nonparametric test followed by Dunn’s multiple comparison post-test, ns: no significance). (C) Compared with SD + Saline, COG1410 reduced the mean microglial branch length in the hippocampus (*n* = 60 cells from 8 samples per group, nonparametric test followed by a Dunn’s multiple comparison post-test, ns: no significance, ** *p* < 0.01, **** *p* < 0.0001). (D) Compared with SD + Saline, COG1410 reduced the mean microglial branch length in the hippocampus (*n* = 60 cells from 8 samples per group, nonparametric test followed by Dunn’s multiple comparison post-test, ns: no significance, ** *p* < 0.01, **** *p* < 0.0001). (E) Compared with SD + Saline, COG1410 increased the microglial total terminals in the hippocampus (*n* = 60 cells from 8 samples per group, nonparametric test followed by Dunn’s multiple comparison post-test, ns: no significance, *** *p* < 0.001, **** *p* < 0.0001). (F) Compared with SD + Saline, COG1410 increased the microglial total branch points in the hippocampus (*n* = 60 cells from 8 samples per group, nonparametric test followed by Dunn’s multiple comparison post-test, ns: no significance, *** *p* < 0.001, **** *p* < 0.0001). (G) Quantitative analysis of the intersections of projections in the Sholl analysis (*n* = 60 cells from 8 samples per group, nonparametric test followed by Dunn’s multiple comparison post-test, CTRL + Saline vs SD + Saline: ** *p* < 0.01, *** *p* < 0.001, **** *p* < 0.0001; SD + Saline vs SD + COG1410: # *p* < 0.05).

After morphological analysis, we performed functional analysis of hippocampal microglia. As CD86 and CD206 are widely regarded as markers of M1/M2 polarization in microglia,^37,38^ we examined the expression of CD86 and CD206 by immunofluorescence staining. An increase of CD86 expression and a decrease of CD206 were observed in sleep-deprived mice’s hippocampal microglia, while COG1410 could reverse the above changes developed in SD mice (Figure S3A–F). These results suggest that SD might promote mouse hippocampal microglia to turn into a pro-inflammatory state, but COG1410 could transform the pro-inflammatory state of microglia into an anti-inflammatory state. This is why COG1410 could restore the relevant impairment of long-term spatial memory resulting from SD.

### 2.7 Protection of COG1410 on Long-Term Memory Loss Induced by Chronic SD Was Weakened by Knockdown of TREM2 in the Hippocampus

To further demonstrate the protective effect of COG1410 on SD mice, a TREM2 small interfering RNA (TREM2-siRNA or Si-TREM2) or a nonspecific siRNA (NS-siRNA or Si-NC) was injected into the bilateral hippocampus every 3 days from day 1 to stably downregulate the expression of TREM2. Then, hippocampal tissue was collected for western blot analysis on day 12 (Figure 7A).

**Figure 7:**
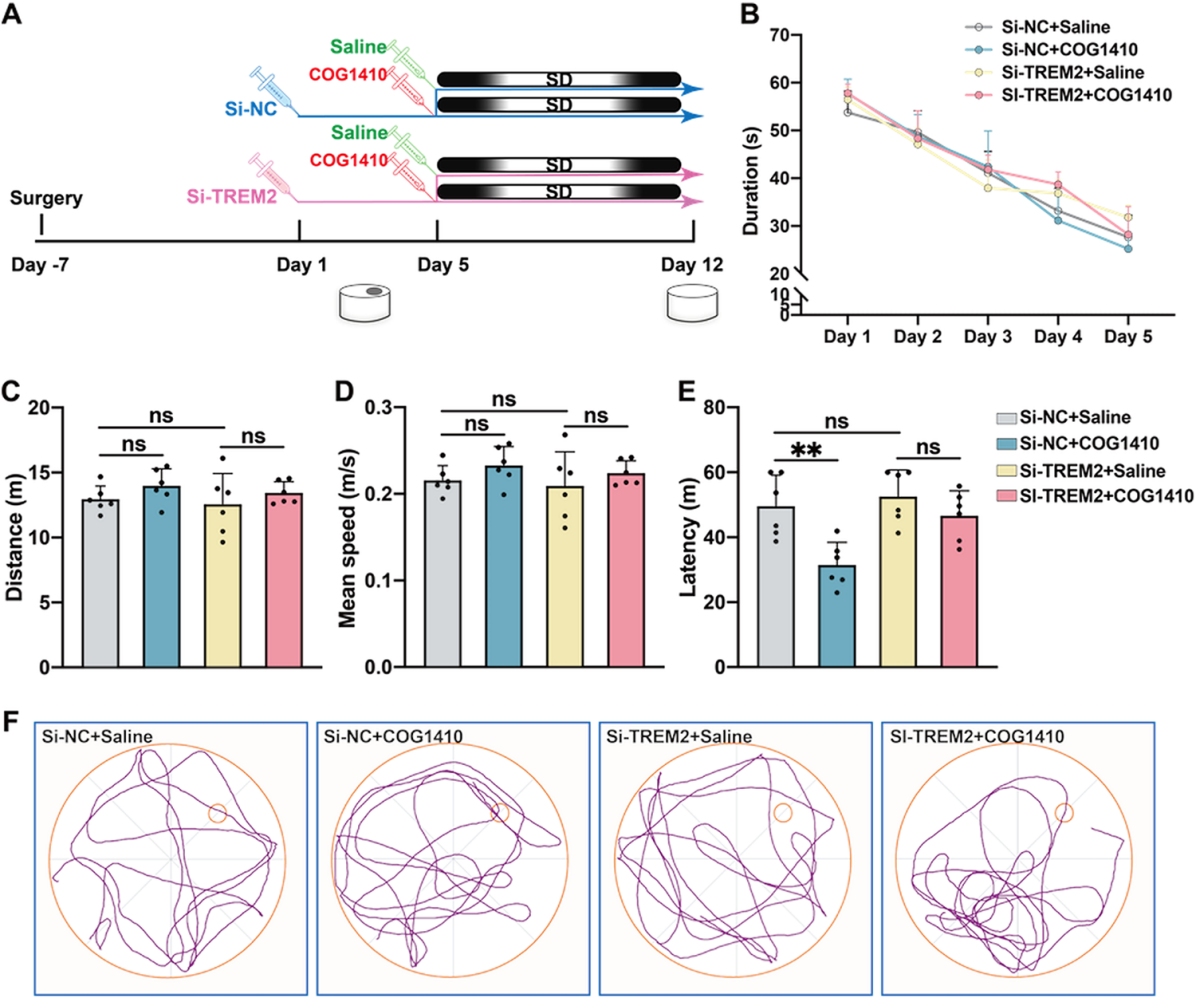
Downregulation of TREM2 weakens the protective effect of COG1410 on long-term memory loss in SD mice. (A) Experimental design: TREM2-siRNA or NS-siRNA mice were injected into the hippocampus every 3 days from day 1, and COG1410 or saline was injected daily intraperitoneally 30 min before SD from day 5 to day 12. (B) Duration of the mice in spatial learning (*n* = 6 per group, two-way ANOVA test followed by Tukey’s multiple comparison post-test, ns: no significance). (C–D) No significant difference was observed between the four groups in swimming distance or swimming speed in the probe test (*n* = 6 per group, one-way ANOVA test followed by Tukey’s multiple comparison post-test, ns: no significance). (E) After 7-day SD, COG1410 reduced the latency of Si-NC mice in the probe test but did not reduce the latency of Si-TREM2 mice (*n* = 6 per group, one-way ANOVA test followed by Tukey’s multiple comparison post-test, ns: no significance, ** *p* < 0.01). (F) Representative track plot of four groups of mice in the probe test.

During spatial learning, mice injected with NS-siRNA or TREM2-siRNA showed similar learning curves during MWM (Figure 7B). After 7 days of SD, the four groups of mice showed similar athletic abilities in the probe test (Figure 7C–D). In the two NS-siRNA groups, compared with the Saline group, COG1410 reduced the latency for SD mice to enter the platform region; however, in the two TREM2-siRNA groups, COG1410 did not reduce the latency in SD mice when compared to the Saline ones (Figure 7E). Thus, our results support that the protective effect of COG1410 on long-term memory loss induced by SD depends on TREM2 expression in the hippocampus.

## 3 Discussion

Given the increasing prevalence of sleep loss in modern society and its detrimental cognitive consequences, we hope to find a solution to alleviate its related cognitive impairment after determining the true mechanism of SD-induced nerve damage in a mouse model. In this study, we used a 7-day MMPM mouse model to explain the impairment of chronic sleep deprivation induced by cognitive processing. We found that chronic sleep deprivation could injure mice’s memories formed before SD but not their working memory after SD. Regarding the molecular mechanism, we discovered that hippocampal microglia exhibited significant downregulation of their phagocytic activity post-SD, concomitant with elevated synaptic density in hippocampal neurons. In further study, we observed that both the above animal phenotypic changes and cellular responses induced by SD could partially be restrained by treatment with APOE analogue COG1410 (preadministration before SD). Finally, downregulation of hippocampal TREM2 expression with siRNA reduced the protective effect of COG1410 on SD and induced long-term memory loss.

The relationship between sleep and memory has been extensively investigated in neuroscience research. The prevailing view is that sleep deprivation has a negative impact on memory,^39,40^ but no final conclusions have been reached on its deep mechanism. Existing studies predominantly focus on SD-induced memory accuracy deficits; detail opinions including: 6h-SD disrupted the memory of mice in the fear condition experiment,^41^ and subacute or chronic sleep deprivation (72 h-SD or 7 d-SD) disrupted the learning ability of mice in MWM.^42–44^ The MWM, a validated behavioral paradigm assessing spatial learning and memory in rodents through navigation challenges,^45^ has become a standard metric in SD research. To specifically examine SD’s impact on pre-established memory consolidation, our experimental design strategically positioned spatial learning prior to SD induction, followed by post-SD probe testing. Consistent with the existing literature, our results revealed significant impairment in spatial memory consolidation among SD mice, as evidenced by reduced target quadrant preference and prolonged platform localization latency during probe tests, despite locomotor activity comparable to controls. Notably, working memory capacity remained intact in SD subjects, contrasting with certain previous reports.^28^ After the detail is canvassed, this discrepancy may stem from SD paradigm specificity: Unlike an automated cylindrical apparatus that could achieve total sleep deprivation, including rapid eye movement (REM) and non-REM (NREM), our MMPM-based protocol primarily disrupted REM sleep. Given the unresolved relationship between REM/NREM deprivation and working memory regulation, further mechanistic investigations are required to clarify the differential effects.

During sleep, the animal’s body is usually unresponsive to the outside world, but the central nervous system remains active,^40^ and it is the activity of the brain that determines whether memories are consolidated or forgotten.^39^ The structural foundation of these cognitive processes lies in neuronal networks through dynamic dendritic spine remodeling. Experimental evidence demonstrates that sleep facilitates memory encoding through motor cortex-mediated new dendritic spine formation,^46^ while REM sleep orchestrates selective synaptic pruning to optimize memory consolidation.^47^ To investigate SD’s neuroplastic effects, we focused our attention on the effects of SD on dendritic spines and their synapses. Our findings revealed increased dendritic spine density in SD-exposed mice, aligning with reports of sleep loss-induced spinogenesis in hippocampal neurons.^7,48,49^ This contrasts with opposing reports documenting spine density reduction under similar conditions.^50–52^ However, the quantity of dendritic spines is not equal to their quality because dendritic spines can be classified as mature or immature according to their morphology. We believe the reason for our opposite observation in sleep-deprived mice is that the increased dendritic spines induced by SD were newly added spines that had not yet formed mature and stable synaptic structures. Regrettably, the imaging resolution of our equipment is limited, and it is unable to provide morphological identification of dendritic spines. Therefore, we evaluated the maturity of the hippocampal synapses through the expression of PSD-95, which is a membrane protein that regulates synaptic strength.^53^ Consistent with most studies, the expression of PSD-95 in our experimental mice decreased after sleep deprivation,^54,55^ which indicated that sleep deprivation decreased the maturity of hippocampal synapses.

In recent years, the mechanisms of damage to neurons and synapses have received much attention in the study of sleep deprivation.^56–58^ Also, we explored the molecular mechanism of SD impairment. Microglia, resident immune cells of the CNS, serve as dynamic regulators of synaptic plasticity and preferentially respond to changes in the whole environment of the brain through their surveillance and phagocytic functions.^55,59^ Our experimental paradigm first assessed hippocampal microglial density post-SD, revealing no significant change in number, a finding congruent with Tuan et al.’s observations.^48^ Subsequently, shifting focus on exploring whether the functional capacity of microglia was impacted by SD. In view of the important role microglia play in pruning excess synapses, which is the guarantee of neurodevelopment,^54,56^ we evaluated synaptic pruning efficacy. Rationally, downregulation of CD68, a lysosomal phagocytic marker in hippocampal microglia, confirmed our expectation. This aligns with current models proposing that sleep deprivation damages memory consolidation by injuring the phagocytic capacity of microglia.^7,48,57^

TREM2, a receptor specifically expressed on the microglial surface, has been demonstrated to play a crucial part in learning and memory by modulating synaptic pruning during CNS development.^18^ In our study model, the expression of TREM2 in hippocampal microglia did not change after SD, though it was reported to be downregulated after chronic SD.^28,29^ Intriguingly, we observed a significant downregulation of its cognate ligand APOE in the hippocampus of SD mice. The level of APOE in the hippocampus was impacted by sleep deprivation; therefore, we asked whether the APOE mimic peptide COG1410 would modulate microglial capacity of engulfing dendritic spines to alleviate SD-induced memory loss. Besides, downregulation of hippocampal TREM2 reduced the protective effect of COG1410 on long-term memory loss in an SD mouse model. Thus, we proved that preventive administration of COG1410 significantly ameliorated spatial memory deficits in SD mice through the Morris water maze (MWM) test. Mechanistically, COG1410 showed the possibility of preserving synaptic integrity in SD mice. Furthermore, we detected that neuroprotection by COG1410 was mediated through the restoration of microglial phagocytic competence rather than promoting the release of the cell factor BDNF. Last but not least, COG1410 targets TREM2 to exert the above protective effect. Excitingly, the above results were consistent with previous research that COG1410 could inhibit the activation of microglia and played a role in neural protection in multiple models.^23,30,32,33,60^

Microglial morphology is related to its functions.^61^ Consistent with established observations, chronic SD induced activation in microglial morphology, such as the reduction of branches and cell retraction. Complementing these morphological shifts, we detected upregulated CD86 (an M1 polarization marker) concomitant with downregulated CD206 (an M2 marker),^38^ collectively indicating SD-induced microglial polarization toward a pro-inflammatory phenotype. COG1410, as an anti-inflammatory substance, also effectively regulates the microglial inflammatory state in chronic SD models. However, whether inflammatory state modulation directly regulates microglial capacity for mature synapse elimination under SD conditions warrants further research.

## 4 Conclusions

In our study, we found that chronic sleep deprivation weakened the hippocampal microglial capacity for synaptic phagocytosis, leading to an increase of immature synapses, which disrupted long-term memory consolidation in mice. COG1410 (APOE mimic peptide, targeting TREM2) could restore the phagocytosis of microglia and alter the microglial polarization state, which could alleviate the memory accuracy in SD mice. In summary, APOE mimic peptide TREM2 is a promising drug target to relieve memory injury resulting from sleep deprivation, but more evidence still needs to be collected to demonstrate the effect of the APOE mimic peptide on memory impairment induced by sleep loss in human beings.

## 5 Methods

### 5.1 Animals

Male mice of C57BL/6 J background (8–10-week old, 22–25 g) were obtained from Guangdong Medical Laboratory Animal Center (Guangdong Province, China) and housed under a 12 h/12 h light/dark cycle in a pathogen-free animal facility with free access to food and water. All animal procedures were approved by the Animal Ethics Committee of the Guangdong Laidi Biomedical Research Institute (Guangzhou, China).

### 5.2 Chronic SD Mice Model

Similar to our previous research, chronic SD was induced using a modified multiple-platform method in a water tank.^62^ Briefly, SD mice were placed in a box with several small platforms (diameter 3 cm), 0.5 cm above the water surface. When mice slept, they awoke because they fell into the water. In contrast, CTRL mice were placed in a box with a large platform (0.5 cm above the water surface) where they would get full sleep. Food and water were freely available to each group. The SD procedure lasted 16h per day (from 20:00 to 12:00 the next day) for 7 days.

### 5.3 Behavioral Test

All behavioral experiments were conducted in a dedicated room during the day (13:00–17:00). Mice were first moved to the test room to adapt to the experimental environment 1 h before the test.

### 5.4 Morris Water Maze (MWM)

The Morris water maze test was carried out in a tank with a diameter of 1.2 m. The tank was artificially divided into four quadrants, and an 8 cm hidden platform was placed in the middle of the NE quadrant, 0.5–1 cm below the water surface during the learning stage. The entire MWM program was divided into 5 consecutive days of spatial learning and a probe test after SD. Spatial learning was conducted 4 times a day from day 1 to day 5 to obtain a solid spatial memory, and a probe test was conducted after SD to evaluate the effect of SD on memory. During the probe test, the platform was removed. A video tracking system (ANY-maze, Stoelting, USA) was used to capture data, and the swim speed, swim distance, latency to find the platform, and time within the targeted quadrant were recorded for statistical analysis.

### 5.5 Novel Object Recognition Test (NORT)

Working memory was assessed using the novel object recognition test (NORT). A box (25 cm × 25 cm x 40 cm) was used for testing. The NORT was divided into two phases. During the training stage, two 3 cm diameter cylinders were placed on either side of the box, allowing the mice to explore freely for 10 min to familiarize themselves with the object. In the test phase, one of the cylinders was replaced with a cuboid (3 × 3 cm), allowing the mice to freely explore the box for 5 min. A mouse’s nose within 2 cm of an object was recorded as exploring. The travel distance, time taken to explore the new object, and total time taken to explore the two objects were recorded and analyzed. The recognition index (RI) was calculated according to the following formula.

RI(%)=[time(new)]/[time(new)+time(old)]×100

### 5.6 Stereotaxic Surgery and siRNA Injection

Seven days before spatial learning, two catheters (RWD, 27 g/M3.5, China) were implanted to the bilateral hippocampus of mice (AP = −2 mm, ML = ± 1.8 mm, DV = 2 mm) with a gripper and followed by cobering the skull surface with dental cement.^62^ On days 1, 4, 7, and 10, a total of 1.0 ul TREM2-siRNA or NS-siRNA solution^63^ (5 nmol) was injected into the bilateral hippocampal region of each mouse using a microsyringe pump with an injection speed of 0.2 ul/min. After injection, the needle was retained for 2 min to ensure that the solution diffused fully.

The sequences of siRNAs were

control siRNA: forward 5′-UUCUCCGAACGUGUCACGUTT-3′,

reverse 5′-ACGUGACACGUUCGGAGAATT-3′;

TREM2-siRNA: forward 5′-GGAGGUACGUGAGAGAAUUTT-3′,

Reverse 5′-AAUUCUCUCACGUACCUCCTT-3′

### 5.7 Brain Acquisition and Section Preparation

After all behavioral tests, the mice were anesthetized and perfused transcardially with cold PBS. Mouse brains were extracted and divided into two sides along the cerebral line. Hippocampal tissue was carefully dissected randomly from one side of the brain and placed in a −80 °C refrigerator for western blotting. The other side of the brain was stored overnight in 10% formalin at 4 °C. Then, 10%, 20%, and 30% gradient dehydration were used in turn. Brain samples were cut into 30 μm-thick sagittal sections containing the hippocampus using a cryotome (CM1950, Leica, Germany). The sections were stored in a −80 refrigerator until further analysis.

### 5.8 Nissl Staining

Nissl staining was carried out according to the manufacturer’s instructions. Briefly, the sections were rinsed twice with distilled water for 2 min each time. Incubation was performed at 37 °C for 5 min with Nissl staining solution (C0117, Beyotime, China). Finally, after dehydration and transparency, the sections were sealed with neutral gum and observed under a bright-field microscope immediately.

### 5.9 Western Blotting

Hippocampal tissues were lysed in RIPA lysis buffer with PMSF and phosphatase inhibitor cocktail. The protein supernatant was assessed with a BCA assay kit, separated on 10% SDS-PAGE gels, transferred to PVDF membranes, and then blocked at room temperature for 2 h with 5% nonfat milk. The membranes were incubated at 4 °C overnight with the following primary antibodies: mouse anti-PSD-95 (1:1000, MA1-046, ThermoFisher), rabbit anti-CD68 (1:1000, #97778, Cell Signaling Technology), rabbit anti-BDNF (1:1000, 28205-1-AP, Proteintech), rabbit anti-TREM2 (1:500, 27599-1-AP, Proteintech), rabbit anti-APOE (1:2000, A16344, Abclonal), rabbit β-tubulin antibody (1:5000, AC015, ABclonal), or rabbit GAPDH antibody (1:5000, AC015, ABclonal). The membranes were washed with TBST and incubated with horseradish peroxidase-conjugated sheep antirabbit secondary antibody (1:5000, A0208, Beyotime) or horseradish peroxidase-conjugated sheep antimouse secondary antibody (1:5000, A0216, Beyotime) for 2 h at room temperature. Finally, the membranes were developed using the Enhanced Chemiluminescence (ECL) Plus detection system.

### 5.10 Golgi-Cox Staining

Mice were sacrificed by cervical dislocation, and brain samples were isolated and washed with PBS. Brain samples were processed with the FD Rapid GolgiStain Kit according to the manufacturer’s instructions. The coronary sections of the brain containing the hippocampus were obtained by CM-1950 (Leica, Germany) with a thickness of 100 um and continuously photographed with BX-X810 at 1 um intervals along the *Z*-axis under the bright field. Individual neurons were selected for quantification of dendritic spines with ImageJ (NIH). The results are shown as the average density of at least 5 dendrites per cell.

### 5.11 Immunofluorescence Staining

Sections of 30 um thick were transferred to PBS blocking solution containing 5% bovine serum albumin and 0.5% Triton X-100 for 2h. After blocking, sections were incubated at 4 °C overnight with the following primary antibodies:, mouse anti-Iba-1 (1:750, ab283319, Abcam), rabbit anti-NeuN (1:500, ab245227, Abcam), rabbit anti-CD68 (1:200, #97778, Cell Signaling Technology), mouse anti-PSD-95 (1:100, MA1-046, ThermoFisher), rabbit anti-CD86 (1:200, 13395-1-AP, Proteintech), or rabbit anti-CD206­(1:200, 18704-1-AP, Proteintech). The goat antimouse IgG H&L (Alexa Fluor 594) (1:500, ab150116, abcam) and goat antirabbit IgG H&L­(Alexa Fluor 488) (1:500, ab150077, abcam) were incubated with the sections for 2 h at room temperature in the dark. The nuclei were stained with DAPI (R37606, ThermoFisher).

Immunofluorescent images were continuously captured with BX-X810 at 0.5 um intervals along the *Z*-axis and superposed to become one clear image. The density of microglia was obtained by manually counting Iba-1 and DAPI double-positive cells and dividing by area. The number of pixels per image with intensity above a predetermined threshold level was considered as a positively stained area for a marker of interest and quantified using ImageJ. The results are shown as the percentage of the positive area to the total area or positive fluorescence intensity.

### 5.12 Sholl Analysis and Microglial Morphology Analysis

Sholl analysis was used to quantitatively analyze the morphology of microglia and was reported as a reflection of microglial activation.^7,61^ The multiple-layer scanning images of Iba-1 were used for analysis. Microglia whose soma was located in the middle of the section were selected because their complete form was preserved as far as possible. The plugin Simple Neurite Tracer (SNT 4.2.1, ImageJ) was used to manually trace the microglial branches from the central soma, and the following data were recorded: the longest branch length, the mean branch length, the total branch length, the number of terminals, the number of branch points, and the number of intersections per radius (increased by 5 μm).

### 5.13 3D Colocalization Analysis and Microglial Engulfment

For the internalization of PSD-95 in microglial lysosomes, sections were taken as continuous multilayer scanning images at an interval of 0.1 μm along the *Z*-axis to confirm the colocalization of PSD-95 and CD68 in the hippocampus. The volume of PSD-95 + CD68 and the volume of CD68 were calculated by a 3D object counter in ImageJ. The ratio of microglial phagocytosis of synapses was calculated using the following formula:

$$\mathrm{PSD}‐95\;\mathrm{content}\;\left(\%\right)=\mathrm{volume}\left(\mathrm{CD}68^{+}+\mathrm{PSD}‐95^{+}\right)/\mathrm{volume}\left(\mathrm{CD}68^{+}\right)\times 100$$

### 5.14 Statistical Analysis

GraphPad Prism software v8.4 was used for statistical analysis. Unpaired 2-tailed Student’s *t-*test, Welch’s *t* test, and Mann–Whitney U test were used to examine the statistical significance between the 2 groups. For comparison of multiple groups, one-way ANOVA or a nonparametric test was used, followed by Tukey’s or Dunn’s posthoc test. All data are presented as mean ± SEM, and *p* < 0.05 was considered statistically significant.

## Supplementary Material



## Notes

COG1410 did not change the motor ability in the probe test (Figure S1) or the expression of BDNF in the hippocampus of SD mice (Figure S2); COG1410 alleviates the SD-induced M1 polarization of hippocampal microglia (Figure S3); TREM2-siRNA downregulates the expression of TREM2 in hippocampus; and original western blot (WB) images (PDF)

## Abbreviations

APOE: apolipoprotein E
BDNF: brain-derived neurotrophic factor
MWM: Morris water maze
NORT: novel object recognition test
SD: sleep deprivation
TREM2: triggering receptor expressed on myeloid cells-2
PSD-95: postsynaptic density protein-95.
CNS: central nervous system
