# Supplementary material for: COG1410 Alleviated Chronic Sleep Deprivation-Induced Memory Loss by Regulating Microglial Phagocytosis and Inhibiting Hippocampal Inflammation
Source: ACS Chem Neurosci. 2025 Jul 9;16(15):2921–34. doi: 10.1021/acschemneuro.5c00214 (PMC12333007; doi:10.1021/acschemneuro.5c00214)
Supplement: Supplementary file 1 [file cn5c00214_si_001.pdf]

Title page

Article Title: COG1410 Alleviated chronic Sleep-Deprivation induced Memory Loss by Regulating Microglial Phagocytosis and Inhibiting Hippocampal inflammation

Author names: Peng Wu[1], Chao Fu[1,2], Min Chen[3], Fanchan Wu[4], Wanyou He[4], Qichen Luo[1], Hanbing Wang[4,**], Yalan Li[1,*]

[*] Co-corresponding author

[**] Corresponding author

Author address

1. Department of Anesthesiology， Jinan University First Affiliated Hospital, 613# The West of Huangpu Avenue, Tianhe District Guangzhou, 510630 Guangdong, China.

2. Department of Anesthesiology, The Second Affiliated Hospital of Guangzhou University of Chinese Medicine, 111# Dade Road, Yuexiu District, Guangzhou, 510120 Guangdong, China.

3. Department of Cardiovascular, Department of Cardiovascular, Guangdong Provincial Key Laboratory of Coronary Heart Disease Prevention, Guangdong Cardiovascular Institute, Guangdong Provincial People's Hospital (Guangdong Academy of Medical Sciences), Southern Medical University, 106# Zhongshan Second Road, Yuexiu District, 510080, Guangzhou, China.

4. Department of Anesthesiology， The First People's Hospital of Foshan, 81# North of Ling Nan Road Foshan, 528000 Guangdong, China.

Corresponding authors

* Corresponding author: Yalan Li, Department of Anesthesiology, Jinan University First Affiliated Hospital, 613# The West of Huangpu Avenue, Tianhe District Guangzhou, 510630 Guangdong; China; E-mail: tyalan@jnu.edu.cn

** Co-corresponding author: Hanbing Wang, Department of Anesthesiology, The First People's Hospital of Foshan, 81# North of Ling Nan Road Foshan, 528000 Guangdong; China; Email: fswhbing@126.com

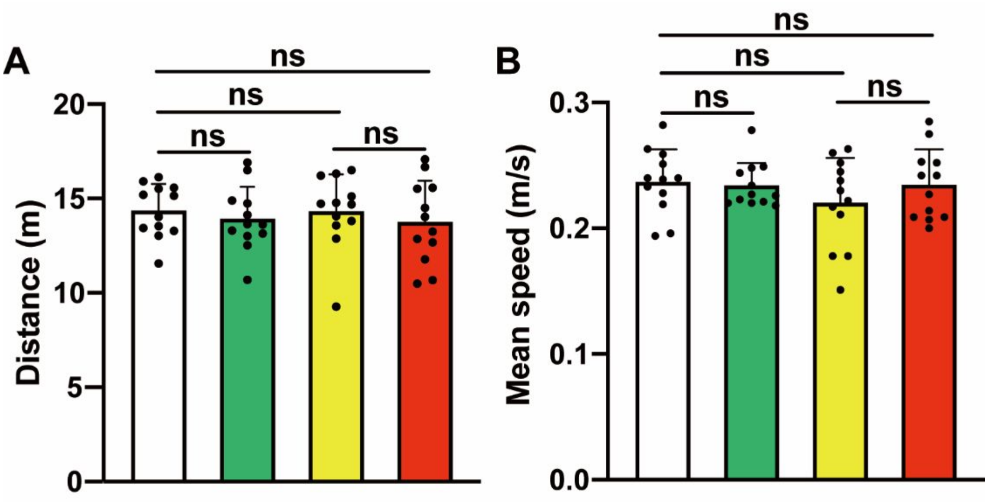

Figure S1. COG1410 didn't change the motor ability in probe test.

A. Compared with SD+Saline, COG1410 didn't affect the swimming distance in probe test. (n=12 per group. Nonparametric test and followed by a Dunn's multiple comparison post -test, ns no significance). **B.** Compared with SD+Saline, COG1410 didn't affect the swimming speed in probe test. (n=12 per group. Nonparametric test and followed by a Dunn's multiple comparison post -test, ns no significance).

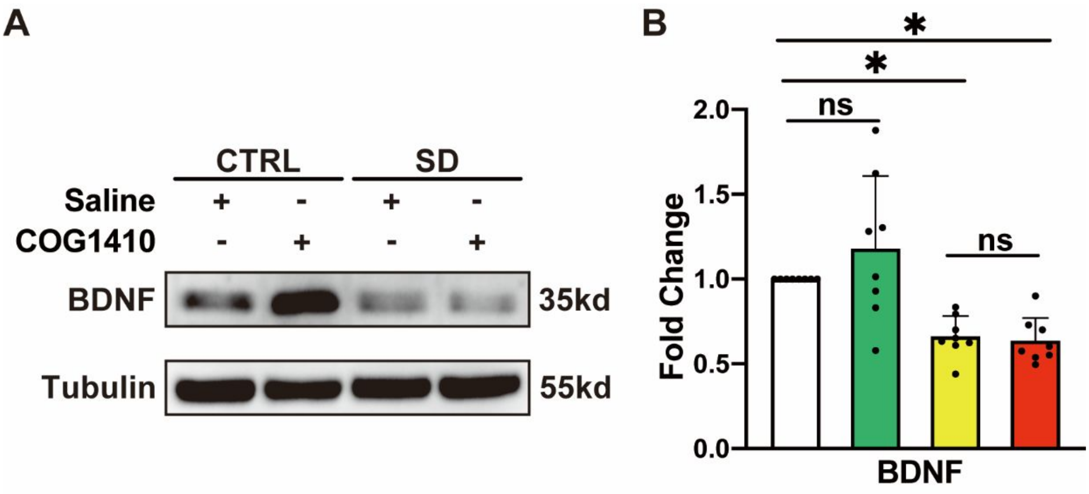

Figure S2. COG1410 didn't change the expression of BDNF in hippocampus of SD mice.

A. Representative images of the expression of BDNF in hippocampus. **B.** Compared with SD+Saline, COG1410 increased the expression of BDNF in hippocampus. The ratio of the BDNF protein to the internal control in the CTRL+Saline group was utilized as a reference for normalization. (n=8 per group. One-way ANOVA test and followed by a Tukey's multiple comparison post-test, ns no significance, * p <0.05, ** p<0.01).

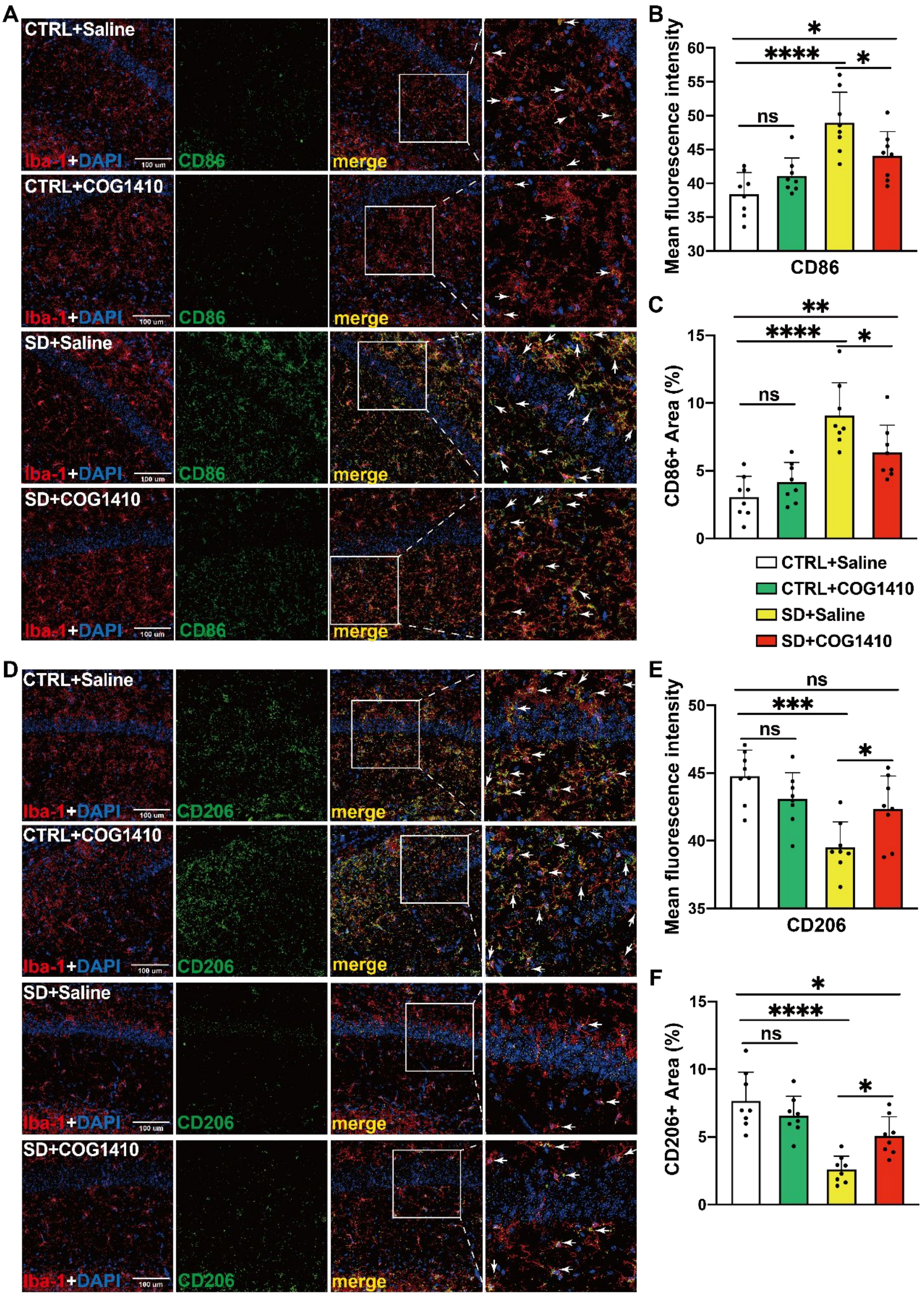

Figure S3. COG1410 alleviates the SD-induced M1 polarization of hippocampal microglia.

A. Representative image of immunofluorescent staining of Iba-1 (red) and CD86 (green) in hippocampus. **B.** Compared with SD+Saline, COG1410 reduced the mean fluorescence

intensity of CD86 in hippocampus. (n=8 per group. One-way ANOVA test and followed by a Tukey's multiple comparison post-test, ns no significance, ns no significance, * p <0.05, **** p <0.0001). **C.** Compared with SD+Saline, COG1410 reduced the CD86 positive fluorescence area in hippocampus. (One-way ANOVA test and followed by a Tukey's multiple comparison post-test, ns no significance, ns no significance, * p <0.05, ** p <0.01, **** p <0.0001). **D.** Representative image of immunofluorescent staining of Iba-1 (red) and CD206 (green) in hippocampus. **E.** Compared with SD+Saline, COG1410 increased the mean fluorescence intensity of CD86 in hippocampus. (n=8 per group. One-way ANOVA test and followed by a Tukey's multiple comparison post-test, ns no significance, ns no significance, * p <0.05, * p <0.05, *** p <0.001). **F.** Compared with SD+Saline, COG1410 increased the CD86 positive fluorescence area in hippocampus. (One-way ANOVA test and followed by a Tukey's multiple comparison post-test, ns no significance, ns no significance, * p <0.05, **** p <0.0001).

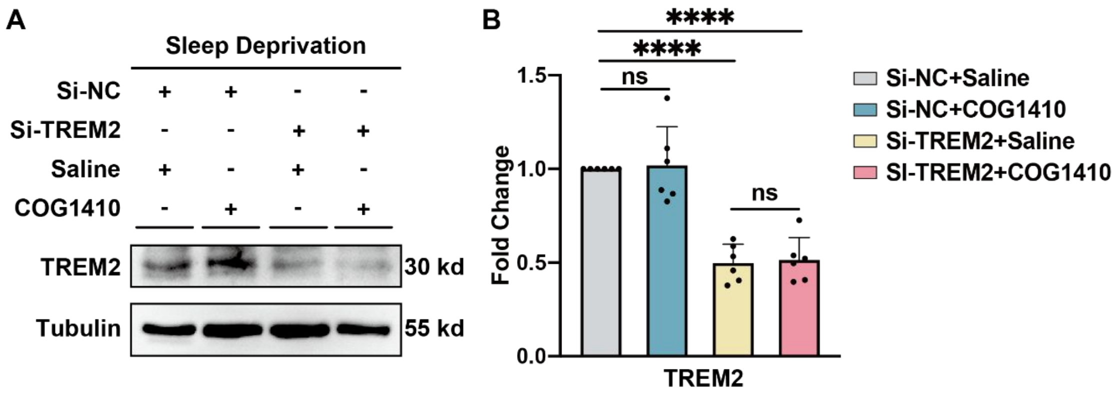

Figure S4. TREM2-siRNA downregulates the expression of TREM2 in hippocampus.

A. Representative images of the expression of TREM2 in hippocampus. **B.**Quantitive western-blot analysis of TREM2 in hippocampus. The ratio of the TREM2 protein to the internal control in the Si-NC+Saline group was utilized as a reference for normalization. (n=6 per group. One-way ANOVA test and followed by a Tukey's multiple comparison post-test, ns no significance, ns, no significance, **** $p<0.0001$).

Fig.2B

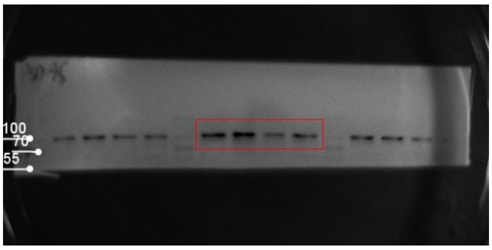

PSD-95

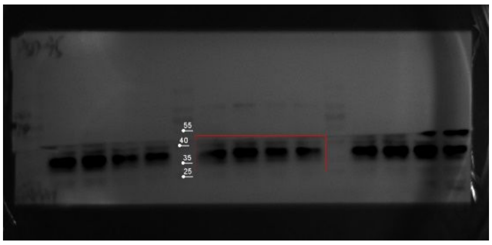

GAPDH

Fig. 3E

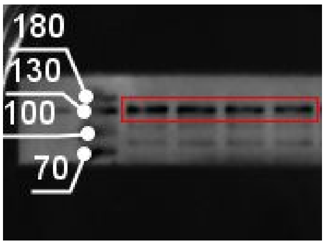

CD68

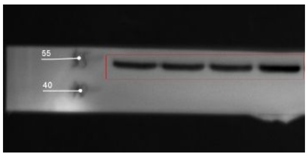

TUBULIN

Fig. 4A

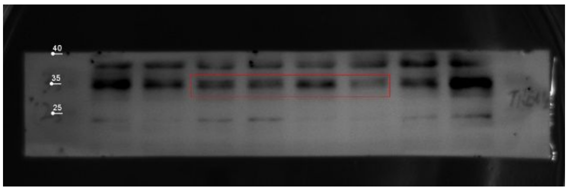

TREM2

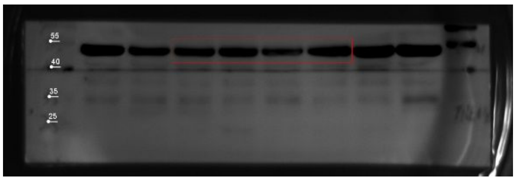

TUBULIN

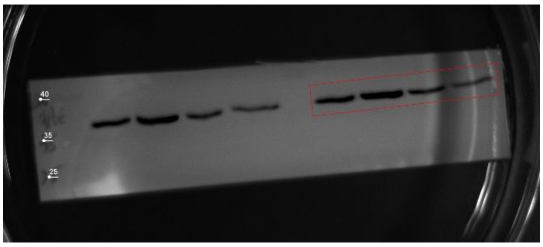

APOE

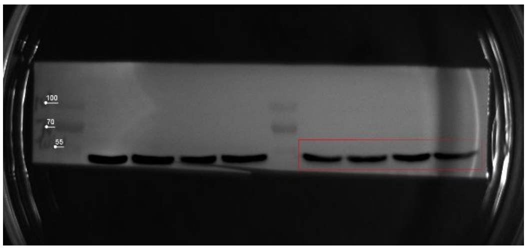

TUBULIN

Fig. 4I

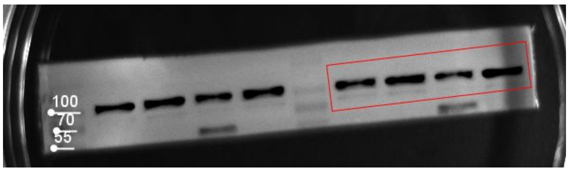

PSD-95

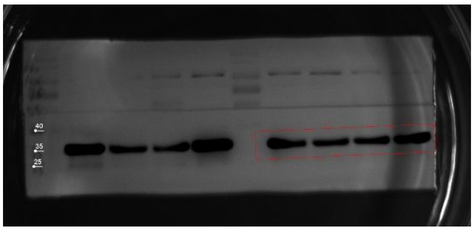

GAPDH

Figure 5D

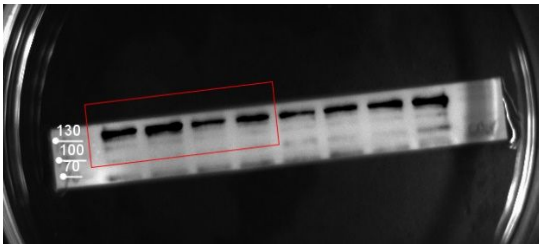

CD68

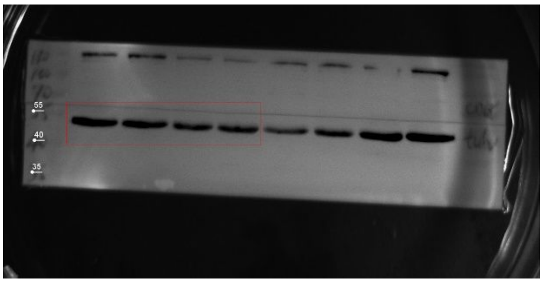

TUBULIN

Figure S2A

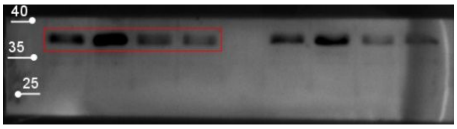

BDNF

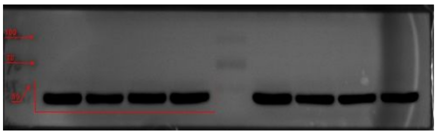

Tubulin

Figure S4A

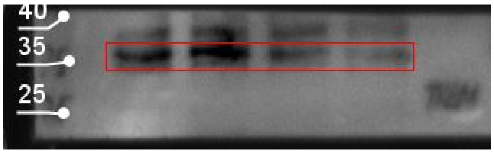

TREM2

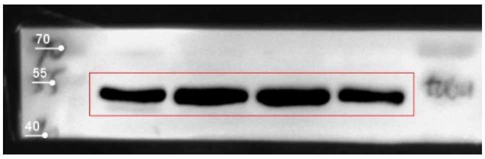

TUBULIN
